# Acanthamoeba castellanii. antibody prevalence among diverse tribal Pakistani population

**DOI:** 10.1186/1742-4690-9-S1-P47

**Published:** 2012-05-25

**Authors:** Abdul Matin, Muhammad Ismail, Khalid Mehmood

**Affiliations:** 1 Institute of Biomedical and Genetic Engineering, Islamabad, Pakistan

## Introduction

Acanthamoeba is opportunistic protozoan pathogen and is known to be one of the most ubiquitous organisms that can produce keratitis and rare but fatal encephalitis. Infections due to Acanthamoeba have increased over the year, which is due to presence of Acanthamoeba in the natural environment and have a direct contact with human in everyday life and is responsible for human diseases. Given the free-living nature of the organisms, it is anticipated that we encounter Acanthamoeba during our normal life. The aim of the present study was to investigate anti-Acanthamoeba antibodies in Pakistani healthy population to combat this pathogen in normal situation.

## Materials and methods

Acanthamoeba isolation from environmental sources (water, soil and air samples) was done using plating assay. Acanthamoeba identification from environmental samples was based on the morphology of cyst and trophozoite forms by non-nutrient agar plates seeded with E. coli K12 and PCR amplification with a genus specific primer pair. The presence of anti-Acanthamoeba sIgA in mucosal secretions of tribal Pakistani population (saliva were obtained from healthy individuals) were determined using Enzyme-linked immunosorbent assays (ELISA).

## Results

Acanthamoeba was successfully isolated from the water sources of Pakistan during this study. ELISA demonstrated the presence of Acanthamoeba-specific sIgA in mucosal secretions of in different age groups and both genders. A total of 524 samples of 45 tribes, were collected from different age groups ranged from 15 to 60 years. The overall prevalence was 78.8% in males and 73.8% in females. No significant difference was observed between genders. The high level of anti-Acanthamoeba antibodies was observed among the people in 25-30 years of age. Furthermore the prevalence of antibodies was observed high in tribal population of Khyber Pakhtunkhwa province as compared to rest of the country.

## Conclusion

Here, we for the first time isolated Acanthamoeba from the natural environment of Pakistan and presented the prevalence level of anti-Acanthamoeba secretory IgA antibody in mucosal secretions of the normal Pakistani population.

